# Effects of probiotics on salivary cytokines and immunoglobulines: a systematic review and meta-analysis on clinical trials

**DOI:** 10.1038/s41598-020-67037-y

**Published:** 2020-07-16

**Authors:** Soraiya Ebrahimpour-Koujan, Alireza Milajerdi, Bagher Larijani, Ahmad Esmaillzadeh

**Affiliations:** 1 Students’ Scientific Research Center, Tehran University of Medical Sciences, Tehran, Iran; 2 Department of Community Nutrition, School of Nutritional Sciences and Dietetics, Tehran University of Medical Sciences, Tehran, Iran; 3 Endocrinology and Metabolism Research Center, Endocrinology and Metabolism Clinical Sciences Institute, Tehran University of Medical Sciences, Tehran, Iran; 4 Obesity and Eating Habits Research Center, Endocrinology and Metabolism Molecular -Cellular Sciences Institute, Tehran University of Medical Sciences, Tehran, Iran; 5 Department of Community Nutrition, School of Nutrition and Food Science, Isfahan University of Medical Sciences, Isfahan, Iran

**Keywords:** Interleukins, Risk factors

## Abstract

Findings on the effects of probiotics on salivary cytokines and immunoglobulines have been conflicting. We aimed to perform a systematic review and meta-analysis on clinical trials that examined the effects of oral intake and local administration of probiotics on salivary cytokines and immunoglobulines in adults. We searched PubMed, MEDLINE, SCOPUS, EMBASE, and Google Scholar up to April 2020 for all relevant published papers assessing probiotic intakes and salivary cytokines and immunoglobulines. We included all randomized clinical trials that investigated the effect of oral probiotic supplementation or lozenges tablets on inflammatory biomarkers in adults. Studies that reported their effect sizes as mean ± SD or mean ± SEM were included. After excluding non-relevant papers, 8 studies remained in this review. Combining findings from 3 studies with 4 effect sizes, we found no significant reduction in salivary IgA concentrations after oral probiotic supplementation [weighted mean difference (WMD): −0.26; 95% CI: (−0.86, 0.35)]. A significant increase in salivary IL-1β concentrations reached after local probiotic supplementation (WMD: 28.21; 95% CI: 18.42, 38.01); however, no significant changes in salivary IL-6 concentrations after local probiotic supplementation was found (WMD: 0.36; 95% CI: −0.85, 1.56). We observed a significant increase in salivary IL-8 concentrations after local probiotic supplementation (WMD: 31.82; 95% CI: 27.56, 36.08). In case of salivary IL-10 concentrations after local probiotic administration, no significant reduction was seen (WMD: −0.02; 95% CI: −0.10, 0.06). we found that oral and local administrations of probiotics might influence some of salivary cytokines. However, additional clinical trials are required to examine these effects on further pro- and anti-inflammatory cytokines and immunoglobulines.

## Introduction

Probiotics have been defined as livings micro-organisms that are selectively fermented^1^. They were reported to have beneficial effects on human health^1,2^. Regular intakes of probiotic supplements alter the gastrointestinal microbiota composition and activity and results in major changes in immune system responses^3^.

Probiotics may influence and enhance innate and adaptive immune response^4^. Several studies have reported the immune-modulatory effects of probiotics in humans^5,6^. Reduction in the production of inflammatory cytokines^7,8^ and elevation of intestinal secretory immunoglobulin A (sIgA) were also reported by probiotics administration^9^. Despite the overall anti-inflammatory effects of probiotics, the potential mechanisms of action are not clearly understood yet. It seems that the stimulatory and regulatory effects of probiotics in immune system confer their immunological protection by changings pro- and anti-inflammatory cytokines profile including TNF-α, IL-1β, IL-6, IL-8 and IL-10^10,11^. In case of their immune-modulatory effects, probiotics beneficially compete with pathogens, nutrients and antagonistic substances, through which they lead to healthy and diverse flora with regulated responses of immune system^12^. Probiotics have been reported to have local (direct) and systemic (indirect) effects on immune system^4^. For instance, they have been involved in maintaining of oral health through inhibiting the growth of pathogens^13,14^. Oral intake of probiotic drinks or supplements enhanced the secretory IgA in saliva^2,6,15^. In addition, local administration of probiotics in lozenges results in higher levels of salivary IgA and specific cytokines^13,14^. However, some other studies failed to find significant changes in salivary immunoglobulines or inflammatory cytokines by either oral intake or local administration of probiotics^2–5,7,15–17^. Despite earlier investigations, there is no comprehensive systematic review or meta-analysis summarizing earlier findings in this regard. We conducted this systematic review and meta-analysis to summarize the available data about the effects of oral intake and local administration of probiotics on salivary cytokines and immunoglobulines in adults.

## Methods

### Search strategy

This systematic review and meta-analysis of clinical trials was conducted based on Cochrane library checklist. All articles published earlier than April 2020 were searched through PubMed, MEDLINE, SCOPUS, EMBASE, and Google Scholar, by two independent investigators to identify relevant articles. To obtain suitable MESH and non-MESH text words, an initial search on Medline was undertaken. The systematic search strategies through each database were provided in the supplementary material file. We had no restrictions of language or time of publication. To avoid missing any publication, a manual search was conducted on reference lists of all included studies as well as review articles. We didn’t include unpublished data and grey literature, including dissertations, thesis, congress papers, and patentsin the current meta-analysis. In addition, duplicate citations were removed.

### Inclusion criteria

We included all randomized clinical trials that investigated the effect of oral probiotic supplementation or lozenges tablets on inflammatory biomarkers in adults. Studies that reported their effect sizes as mean ± SD or mean ± SEM were included. Publications were independently assessed by two reviewers considering the PRISMA (Preferred Reporting Items for Systematic Reviews and Meta-Analyses) checklist. Any disagreements between the reviewers were resolved through discussion.In case of several publications with the same data set, we included only the most complete one^13,16^. If data for specific probiotics were reported separately, we considered them as a separate study in the analysis^1^.

### Exclusion criteria

**Figure 1:**
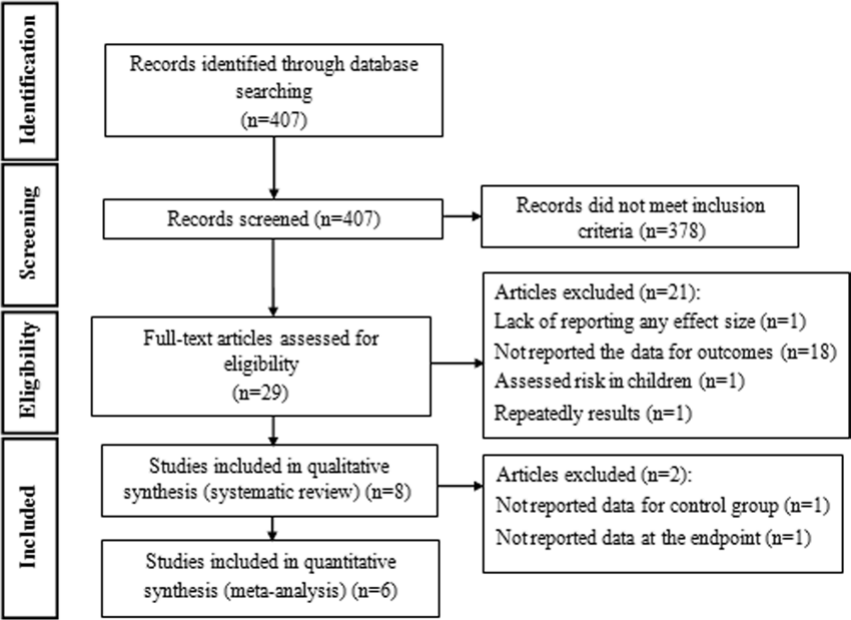
Flowchart of study selection process.

Studies were excluded if they were observational, editorial, letter to editor, comments, ecological or review papers. In addition, studies in which random allocation was not performed, had not control group or those conducted on animal models, pregnant or lactating women, children or elderlies were not included. Publications that examined the effect of another intervention along with probiotic supplementation, those that used symbiotics, examined only gene expression of inflammatory biomarkers or concentrations of inflammatory biomarkers *in-vivo* were not also considered eligible for the current study. Publications that examined gingival index, plaque index, bleeding, depth of pocket and etc. were excluded. The study by Garaiova *et al*. was excluded from systematic review and meta-analysis because its study population was children^18^. We also excluded the study of Dong *et al*. study form the meta-analysis due to not reporting any effect size^3^. In addition, the study of Jorgensen *et al*.^16^ was excluded because the data were repeatedly reported in the study of Braathen *et al*.^13^. After these exclusions, 8 papers remained for the primary systematic review. We didn’t consider two studies in the meta-analysis due not to reporting the data for control group^6^ and in the end of trial for both groups^5^. Figure 1 illustrates the study selection process for systematic review and meta-analysis.

### Data extraction

**Figure 2:**
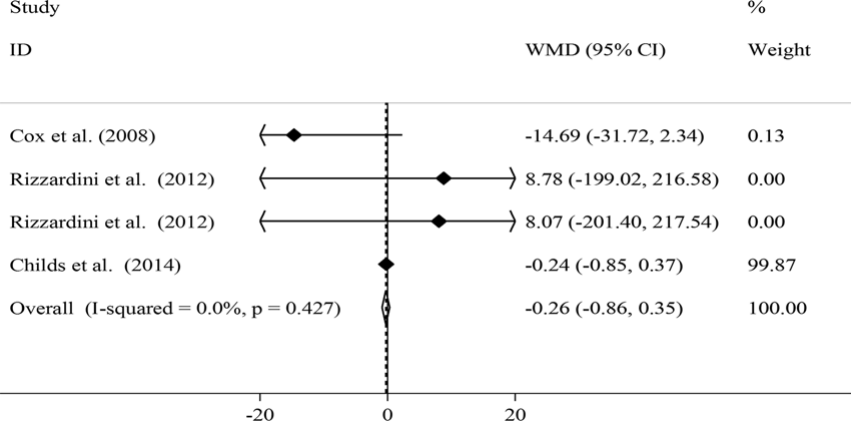
Effects of oral probiotic supplementation on salivary IgA concentrations.

The data were extracted independently and cross-checked by two reviewers (SE and AM). Any disagreements between reviewers were consulted by principal investigator (AE). Quantitative data regarding effect-size measures such as mean and Standard Deviations (SDs) or mean and Standard Errors (SEs) or median and Interquartile Range (IQR) of inflammatory biomarkers before and after intervention in each groups; and mean (SD) changes in inflammatory markers after intervention in each group were extracted.In addition, information on first author’s last name, publication year, subjects’ heath condition, sample size, participants’ sex, number of subjects in each group, participants’ age, type of probiotics, study design (parallel/cross-over/other), type of control, duration of intervention and covariates were obtained. If data were reported as SEs or IQR, they were converted to SDs using appropriate formulas. When the concentration of an inflammatory biomarker was reported in different units, it was converted to the most frequently used one. Three studies had reported results in Figs. 1, 2, 6. We obtained the values from the figures by online “webplot digitizer” converting 2D Bar Plot to data. The values for SD changes were calculated using √S_1_^2^ + S_2_^2^ − 2 × r × S_1_ × S_2_ formula, in which r was computed for each individual study using SD_1_^2^ + S_2_^2^ - SD change^2^/2SD_1_SD_2_. The quality of studies and risk of bias of all eligible studies were assessed using the Cochrane Collaboration’s tool for quality assessment of randomized controlled trials^19^. The quality assessment tool encompasses the following items: random sequence generation, allocation concealment, blinding of participants and personnel, blinding of outcome assessment, incomplete outcome data, selective reporting and other probable sources of biases.

**Figure 3:**
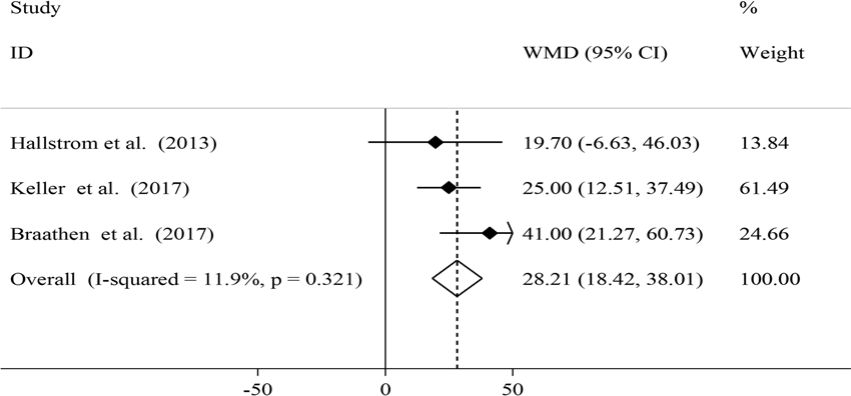
Effects of local probiotic supplementation on salivary IL-1β concentrations.

**Figure 4:**
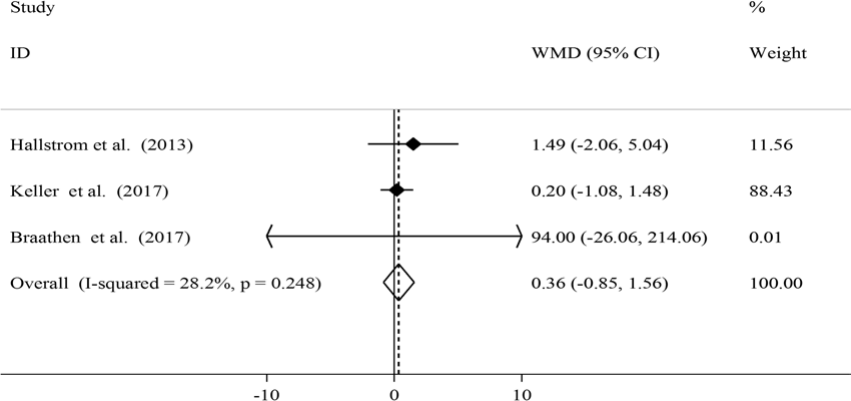
Effects of local probiotic supplementation on salivary IL-6 concentrations.

**Figure 5:**
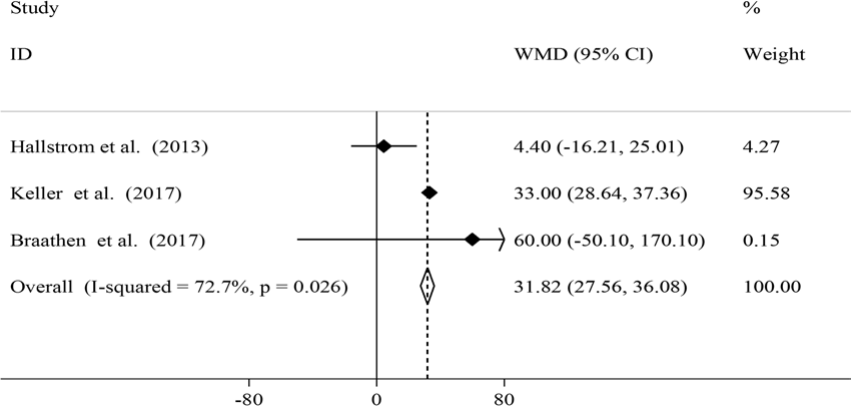
Effects of local probiotic supplementation on salivary IL-8 concentrations.

**Figure 6:**
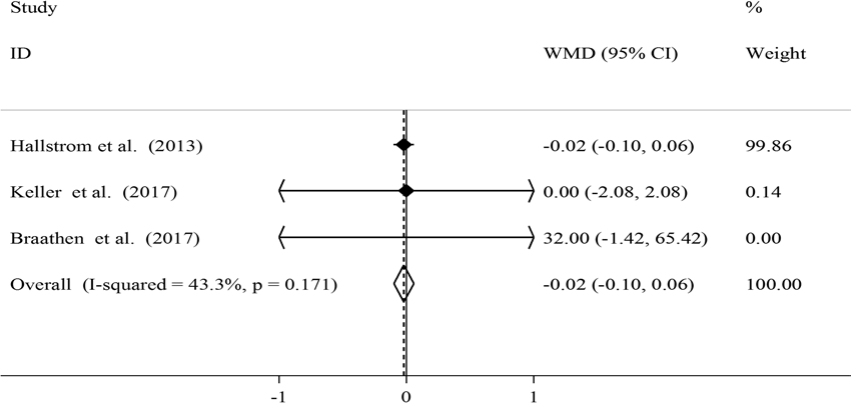
Effects of local probiotic supplementation on salivary IL-10 concentrations.

### Statistical analysis

All effect sizes were calculated as mean ± SD of changes in the concentrations of inflammatory biomarkers between probiotic and control groups. The fixed-effects model was used to calculate the overall effect sizebecause random-effects model gives larger weights to small extreme studies^20^. We examined between-study heterogeneity by the Cochran’s Q test and I^2^ statistic. To find probable sources of between-study heterogeneity, subgroup analyses were conducted based on sex (Male/Female/Both genders), age (<40 year/>40 year), study design (Parallel/Cross-over), supplement dosage (=10^9^/>10^9^ CFU/day), duration of intervention (<3 /≥3 weeks) and probiotic type (Lactobacillus/Bifidobacter/Different types), using a fixed-effects model. The duration of 3 weeks and the dosage of 10^9^ CFU/day were selected based on previous studies^21,22^. All statistical analyses were done using Stata software, version 11.2 (Stata Corp, College Station, TX). *P* < 0.05 was considered as statistically significant.

## Results

### Findings from the systematic review

The initial literature search yielded 407 unique studies. Based on titles and abstracts, 378 studies were excluded. Out of these, 21 studies were also excluded due to above-mentioned reasons. Finally, 8 articles that reported the effects oforal probiotic intake or probiotic containing lozenges tablets on salivary immunoglobulins or cytokines remained for the current study. Main characteristics of five studies that examined the effects of oral probiotic intake on salivary immunoglobulins are presented in Table 1. Five studies were done on healthy adults^1,2,5,6,15^. These studies were published between 2008 and 2016. Except for one study on men^15^, four other studies were performed on both genders. Total sample sizes in intervention and control groups were 231 and 129, respectively (54.92% female and 45.07% male). Participants in these studies were healthy people aged ≥18 years.Three studies were parallel^1,5,6^ and 2 studies were cross-over trials^2,15^. Participants consumed the probiotic supplements or placebos as capsules^1,15^ or milk- or fruit juice-based drinks^2,5,6^. Daily dose of supplementation ranged from 10^9^ to 35 × 10^9^. All studies had control group, except for the study of Harbige *et al*.^6^. Administered probiotics were lactobacillus^1,5,6,15^, bifidobacter^1,2,5^ and propionibacterium^5^. Three studies had used more than one type of probiotic^1,2,16^. Duration of trial ranged from 3 to 6 weeks. Measured outcomes were salivary IgA^1,2,5^, IgA1^6,12^, IgA2^6^, IgG^1^, IgM^1^ and INF-γ^6^. The method of assessment of outcome in all studies was enzyme-linked immunosorbent assay (ELISA). Three studies had reported mean ± SE of salivary immunoglobuline concentrations before and after intervention^6^ or their changes^1,2^. Table 2 presents the results of quality assessment of eligible studies on oral probiotic intake. Two studies had poor quality^5,6^, two had good quality^1,2^ and the remaining one study had fair quality^15^. The risk of bias was attributed to random sequence generation and blinding of outcome assessment in the included studies. Due to limited number of studies, we did not perform subgroup analysis by quality of primary studies.

**Table 1:** Effects of oral probiotic intake on salivary immunoglobulins.

Author (yaer)	Subjects and gender	Age range/ And mean (year)	Design	Intervention type		Bacteria type	Duration (wk/d)	Outcomes	Outcome assessment method	outcome		Any other intervention (from)	Notes about subjects	Adjustment or matching
				Intervention (name and composition)	Control (name and composition)					Intervention mean ± SD and number	Control mean ± SD and number			
Harbige *et al*. (2016)	F: 10 M: 8 Both: 18 Probiotic: 14 Placebo: 4	18–49	CT (clinical trial)	Daily drink with breakfast: two 65mlbottles equivalent intake of 1.3 × 10^10^ live Lactobacillus caseiShirota (LcS).	No treatment	Lactobacillus caseiShirota (LcS)	4 week intervention, 6 week break, followed by 4 week intervention	Salivary IgA1, Salivary IgA2, Salivary INF-γ (For 10 probiotic subjects)	Salivary INF-γ: ELISA Salivary IgA1, 2: radial immunodiffusion assay	SIgA1(mg/mL): Before:0.04 ± 0.13 Week 4:0.04 ± 0.16 Week 10: 0.04 ± 0.15 Week 14: 0.05 ± 0.17 N = 10 SIgA2(mg/mL): Before:0.0 3 ± 0.09 Week 4:0.03 ± 0.11 Week 10: 0.03 ± 0.12 Week 14: 0.03 ± 0.13 N = 10 SINF- γ(mg/mL):NR	No	No	Salivary samples just obtained from 10 probiotic subjects. Subjects were healthy volunteers	No
Childs *et al*. (2014)	F: 22 M: 22 Probiotic:42 Placebo: 41	25–65 43 ± 12	Cross-over	The volunteer were given 2 sachets of daily supplements which powders dissolved in water, milk or fruit juice: Prebiotic (xylo-oligosaccharide, XOS, 8 g/d), Probiotic (Bifidobacteriumanimalis subsp. lactis Bi-07, 10^9^ colony-forming units (CFU)/d), Synbiotic (8 g XOS + 109 CFU Bi-07/d)	The volunteer were given 2 sachets of daily placebo which powders dissolved in water, milk or fruit juice: Placebo. maltodextrin (MDX; Syral)	Bifidobacterium animals subsp. Lactis Bi-07, 10^9^ CFU	21 days	Salivary IgA	enzyme-based colorimetry	SIgA(mg/mL): Change: −0.18 ± 0.50 N = 42	SIgA(mg/mL): Change: 0.06 ± 1.92 N = 41	No	BMI for all subjects: were 25 ± 5 kg/m^2^. Subjects were healthy volunteers Symbiotic: 41 Prebiotic: 42	Sex, age, BMI
Rizzardini *et al*. (2012)	F: 118 M: 93 BB-12 cap: 53 (25/28) Placebo cap: 48 (27/21) L. casei 431: 56 (31/25) placebo drink: 54 (35/19)	20–60	Parallel	Intervention groups consumed a minimum of 10^9^ colony-forming units of BB-12 (capsule) or L. casei 431 (dairy drink) once daily (110 ml).	Placebo groups consumed matched placebo capsule or placebo drink once daily (110 ml).	Bifidobacteriumanimalis ssp. lactis (BB-12) capsule and Lactobacillus paracasei ssp. paracasei (L. casei 431) drink	6 weeks	Salivary IgA, IgG, IgM	salivary IgA were analysed using Human Secretory IgA SIgA ELISA Kit, total salivary IgGand IgM were analysed using the Quantitative Human IgG/IgM ELISA Kit	**BB-12:** Change, SIgA (mg/mL): Change: 57.88 ± 612.98 N = 53 Change, SIgG (U /mL): Change: 2.74 ± 153.02 N = 53 Change, SIgM (U/mL): Change: 1.38 ± 220.66 N = 53 **L. casei 431:** Change, SIgA(U/mL): Change: 59.33 ± 594.73 N = 56 Change, SIgG (U/mL): Change: −4.61 ± 176.15 N = 56 Change, SIgM (U/mL): Change: 4.29 ± 203.53 N = 56	**BB-12:** Change, SIgA (U/mL): Change: 49.1 ± 446.36N = 48 Change, SIgG (U/mL): Change: -5.23 ± 173.94 N = 48 Change, SIgM(U/mL): Change: 6.77 ± 240.12 N = 48 **L. casei 431:** Change, SIgA (U/mL): Change: 51.26 ± 525.08 N = 54 Change, SIgG (U/mL): Change: -1.90 ± 133.99 N = 54 Change, SIgM (U/mL): Change: 0.88 ± 204.55 N = 54	2 weeks after intervention, a seasonal influenza vaccination was given to all subjects.	BMI for subjects: BB-12 cap: 22.8 ± 4.1 Placebo cap: 22.4 ± 3.8 L. casei 431: 24.6 ± 4.3 placebo drink: 22.8 ± 3.6 Subjects were healthy volunteers	No
Cox *et al*. (2008)	F: 0 M: 20 Both: 20	27.3	Cross-over	Intervention group was given 3hard gelatin capsules twice daily with food (L fermentum VRI-003 (PCC), contained a minimum of two billion of Lactobacillus fermentum strain VRI-003)	Placebo group was given identical 3 placebo capsules twice daily with food.	Lactobacillus fermentum strain VRI-003	1 month (28 days) intervention 4 months (14 week)	Salivary IgA, IgA1 and albumin	SIgA and SIgA1: ELISA assay	SIgA (mg/mL): Before:56.0 ± 35.4%Change:29.0 ± 80.7 N = 20 SIgA1 (mg/mL): Before:94.5 ± 63.4%Change:21.3 ± 67.0 N = 20	SIgA (mg/mL): Before:69.2 ± 44.7%Change:27.5 ± 58.9 N = 20 SIgA1 (mg/mL): Before:92.7 ± 34.4%Change: 23.6 ± 64.6 N = 20	No	Subjects were healthy volunteers	No
Kekkonen *et al*. (2008)	F: 45 M: 17 Both: 62 Lactobacillus rhamnosus GG: 13 Bifidobacteriumanimalis ssp. LactisBb12: 16 Propioni-bacterium freudenreichii ssp. Shermanii JS: 17 Placebo: 16	44 23–58	Parallel	The subjects were advised to consume a 250 mL milk-based fruit drink daily for 3 wk containing either: L. rhamnosus GG (ATCC 53103) (LGG) bacteria, on average 6.2 × 107 cfu/mL (daily dose of 1.6 × 1010 cfu); B. animalis ssp. lactis Bb12 (Bb12) bacteria, 1.4 × 108 cfu/mL (daily dose of 3.5 × 10^10^cfu); P. freudenreichii ssp. shermanii JS (DSM 7067) (PJS) bacteria, 1.3 × 108 cfu/mL (daily dose of 3.3 × 1010 cfu)	Control group received a placebo drink without any probiotic bacteria.	Lactobacillus rhamnosus GG (LGG), Bifidobacterium animalis ssp. lactis Bb12 (Bb12), or Propionibacteriumfreudenreichii ssp. shermanii JS (PJS)	3 weeks	Salivary IgA	ELISA assay	**LGG:** SIgA(mg/mL): Before:270 ± 210 After: same before N = 13 **BB-12:** SIgA (mg/mL): Before:400 ± 450 After: same before N = 16 **PJS:** SIgA (mg/mL): Before:280 ± 240 After: same before N = 17	**Placebo:**SIgA (mg/mL):Before:230 ± 140 After: same before N = 16	No	BMI for subjects: 24^18–30^ Subjects were healthy volunteers	No

**Table 2:** Study quality and risk of bias assessment of included studies on oral probiotic intake according to the Cochrane Collaboration’s tool.

Study (year)	Random sequence generation	Allocation concealment	Blinding of participants and personnel	Blinding of outcome assessment	Incomplete outcome data	Selective outcome reporting	Other sources of bias	Overall quality*
Harbige *et al*. (2016)	U	U	U	U	L	L	H	Poor
Childs *et al*. (2014)	L	L	L	L	L	L	U	Good
Rizzardini *et al*. (2012)	L	L	L	U	L	L	L	Good
Cox *et al*. (2008)	U	L	L	U	L	L	L	Fair
Kekkonen *et al*. (2008)	U	U	U	U	L	L	L	Poor

U; unclear risk of bias, L; low risk of bias, H; high risk of bias.

*Good quality: all criteria met; Fair quality: one criterion not met (i.e. high risk of bias for one domain or two criteria unclear); Poor quality: two or more criteria listed as high or unclear risk of bias.

Table 3 provides characteristics of three studies^4,13,14^ that examined the effects oflocal administration of probiotic tablets as lozengeson salivary cytokines and immunoglobulins. These studies were published between 2007 and 2017 and were conducted on both genders except for one study on females^14^. Total sample sizes in intervention and control groups were 93 and 66, respectively (68.79% female and 31.21% male). Participants were healthy people aged ≥18 years. Two studies were cross-over^13,14^ and one study was parallel trial^4^. In these publications, participants were healthy participants^13,14^ or periodontal patients^4^. Daily dose of supplementation ranged from 0.1 × 10^9^ to 3 × 10^9^. The administered probiotics in these papers were various strains of lactobacillus. Duration of trials ranged from 3 weeks to 12 weeks. Measured outcomes were salivary IgA^13^, IL-1β^4,13,14^, IL-6^4,13,14^, IL-8^4,13,14^, IL-10^4,13,14^, IL-18^14^ and TNF-α^4,13,14^. The method of assessment of all these variables was enzyme-linked immunosorbent assay (ELISA). All studies had reported mean ± SD of salivary cytokines and immunoglobuline concentrations before and after intervention. The quality assessment of included studies on local administration of probiotic tablets as lozenges revealed that two studies had fair quality^4,14^ and the remaining one study^13^ had good quality **(**Table 4). Allocation concealment and blinding of outcome assessment were the major sources for risk of bias. Again, due to limited number of studies, we were not able to do subgroup analysis.

**Table 3:** Effects of local administration of probiotic tablets as lozenges on salivary cytokines and immunoglobulins.

Author (yaer)	Subjects and gender	Age range/ mean (year)	Design	Intervention type		Bacteria type	Duration (week)	Outcomes	Outcome assessment method	outcome		Any other intervention (from)	Notes about subjects	Adjustment or matching
				Intervention (name and composition)	Control (name and composition)					Intervention mean ± SD and number	Control mean ± SD and number			
Keller *et al*. (2017)	F: 34 M: 13 Both: 47 Probiotic: 23 Placebo: 24	Probiotic: 26.9 Placebo: 25.7	Parallel	The participants were instructed to take one tablet of in the morning and one in the evening30 min after tooth brushing. The probiotic tablets contained an equal mix of Lactobacillus rhamnosus PB01 DSM14869 and Lactobacillus curvatus EB10 DSM32307 at a total dose of ≤108 cfu/tablet	The placebo tablets were identical in size and composition but without the addition of the probiotic strains.	Lactobacillus rhamnosus PB01 DSM14869 and Lactobacillus curvatus EB10 DSM32307	4 weeks intervention	IL-1β, IL-10, IL-8, IL-6, TNF-α	xMAP technology multiplex immunoassay	IL-1β (pg/mL): Before: 50 ± 125 4 weeks:71 ± 155 IL-6 (pg/mL): Before:6.3 ± 9.8 4 weeks:5.6 ± 14.1 IL-8 (pg/mL): Before: 100 ± 113 4 weeks:74 ± 119 IL-10 (pg/mL): Before:9.2 ± 14.3 4 weeks: 9.9 ± 9.2 TNF-α (pg/mL): Before:2.8 ± 3.3 4 weeks:3.1 ± 6.6	IL-1β (pg/mL): Before:25 ± 41 4 weeks:21 ± 35 IL-6 (pg/mL): Before:4.0 ± 5.4 4 weeks:3.1 ± 4.2 IL-8 (pg/mL): Before:94 ± 88 4 weeks:87 ± 79 IL-10 (pg/mL): Before:7.0 ± 8.7 4 weeks:6.3 ± 8.6 TNF-α (pg/mL): Before:3.1 ± 2.9 4 weeks:3.1 ± 3.7	All participants used fluoride toothpaste (1,100-1,450 mg/kg) on a daily basis	There were no significant differences in the baseline characteristics (age, sex, flow rate, oral hygiene routines) between the two study groups. Subjects were patients	No
Braathen *et al*. (2017)	Both: 47 F: 36 M: 11 Prob: 23 Placebo: 24	18–32 23.9 ± 3.3	Cross-over	The active intervention was twice daily intake of one lozenge containing two strains of the probiotic bacterium L. reuteri Prodentis (DSM 17938 1 × 109 cfu/lozenge and 12 5289 2 × 109 cfu/lozenge). The participants were instructed to ingest either probiotic or placebo lozenges twice daily (morning and evening) for three weeks followed by a three-week wash-out period. Hereafter, the participants crossed-over and received the opposite lozenges twice daily for three weeks. The intervention period terminated with a three-week wash-out period	The placebo lozenges were identical in taste, colour, texture and size but without active bacteria	Lactobacillus reuteri	12 weeks	Salivary IgA, IL-1β, IL-10, IL-8, IL-6, TNF-α	Salivary IgA: ELISA Cytokines: xMAP technology multiplex immunoassay	Salivary IgA (mg/100 mL): Baseline:7.7 ± 4.1 Follow-up:9.3 ± 4.6 IL-1β (pg/mL): Baseline:149 ± 365 Follow-up:166 ± 400 IL-6(pg/mL): Baseline:21 ± 22 Follow-up:100 ± 293 IL-8(pg/mL): Baseline:211 ± 187 Follow-up:262 ± 434 IL-10(pg/mL): Baseline:26 ± 25 Follow-up:38 ± 87 N_B_ = 11 N_F_ = 17	Salivary IgA (mg/100 mL): Baseline: 8.6 ± 5.9 Follow-up:5.5 ± 2.3 IL-1β (pg/mL): Baseline:114 ± 161 Follow-up:90 ± 132 IL-6(pg/mL): Baseline:40 ± 71 Follow-up:25 ± 30 IL-8(pg/mL): Baseline:207 ± 218 Follow-up:198 ± 171 IL-10(pg/mL): Baseline:43 ± 75 Follow-up:23 ± 29 N_B_ = 30 N_F_ = 24	No	Subjects were healthy volunteers	No
Hallstrom *et al*. (2013)	F: 18 Total: 18	38	Cross-over	Lozenges containing two strains of L. reuteri (ATCC55730 and ATCC PTA5289; 1 × 10^8^ CFU of each strain) were taken twice a day during the experimental periods	Lozenges containing placebo were taken twice a day during the experimental periods.	L. reuteri (ATCC55730 and ATCC PTA5289	3 weeks	IL-1β, IL-6, IL-8, IL-10, IL-18, TNF-α	Cytokines determined using the commercial Bio-Plex Cytokine Assay (Bio-Rad Laboratories, Hercules, CA)	TNF-α (pg/mL): Baseline:0.72 ± 0.81 Follow-up:1.45 ± 4.14 IL-1β (pg/mL): Baseline:27.6 ± 22.4 Follow-up:76.6 ± 70.2 IL-6(pg/mL): Baseline:3.77 ± 8.56 Follow-up:5.15 ± 16.2 IL-8(pg/mL): Baseline:80.9 ± 57.7 Follow-up:36.8 ± 34.0 IL-10(pg/mL): Baseline:0.36 ± 0.30 Follow-up:0.43 ± 0.46 IL-18(pg/mL): Baseline:42.3 ± 59.8 Follow-up:98.6 ± 105.7 N = 18	TNF-α (pg/mL): Baseline:0.47 ± 0.30 Follow-up:0.66 ± 1.03 IL-1β (pg/mL): Baseline:31.2 ± 27.7 Follow-up:60.5 ± 65.4 IL-6(pg/mL): Baseline:1.69 ± 1.67 Follow-up:1.58 ± 2.45 IL-8(pg/mL): Baseline:81.9 ± 65.3 Follow-up:33.4 ± 27.5 IL-10(pg/mL): Baseline:0.29 ± 0.20 Follow-up:0.38 ± 0.26 IL-18(pg/mL): Baseline:34.0 ± 47.9 Follow-up:116.2 ± 112.1 N = 18	No	Subjects were healthy volunteers	No

**Table 4:** Study quality and risk of bias assessment of included studies on local administration of probiotic tablets as lozenges according to the Cochrane Collaboration’s tool.

Study (year)	Random sequence generation	Allocation concealment	Blinding of participants and personnel	Blinding of outcome assessment	Incomplete outcome data	Selective outcome reporting	Other sources of bias	Overall quality*
Keller *et al*. (2017)	U	U	L	L	L	L	L	Fair
Braathen *et al*. (2017)	L	L	L	U	L	L	L	Good
Hallstrom *et al*. (2013)	L	U	L	U	L	L	L	Fair

U; unclear risk of bias, L; low risk of bias, H; high risk of bias.

*Good quality: all criteria met; Fair quality: one criterion not met (i.e. high risk of bias for one domain or two criteria unclear); Poor quality: two or more criteria listed as high or unclear risk of bias.

### Findings from meta-analysis

Combining findings from 3 studies^1,2,15^ with 4 effect sizes, we found no significant reduction in salivary IgA concentrations after oral probiotic supplementation [weighted mean difference (WMD): −0.26; 95% CI: (−0.86, 0.35)] (Fig. 2). There were no significant between-study heterogeneity (*I*^2^ = 0.0%, P = 0.427). No particular study had a significant influence on the summary effect in our sensitivity analysis. There was no proof of significant publication bias (Egger’s test: 0.494).

There were 3 clinical trials examining local administration of probiotic tablets as lozenges on salivary IL-1β, IL-6, IL-8 and IL-10^4,13,14^. Combining three effect sizes from clinical trials, we found a significant increase in salivary IL-1β concentration after local probiotic supplementation (WMD: 28.21; 95% CI: 18.42, 38.01) (Fig. 3). There were no significant between-study heterogeneity (*I*^2^ = 11.9%, P = 0.32). No particular study had a significant influence on the summary effect in our sensitivity analysis. There was no proof of significant publication bias (Egger’s test: 0.89).

When we combined three effect sizes, we found no significant change in salivary IL-6 concentrations after local probiotic supplementation (WMD: 0.36; 95% CI: −0.85, 1.56) (Fig. 4). There were no significant between-study heterogeneity (*I*^2^ = 28.2%, P = 0.248) and evidence of significant publication bias (Egger’s test: 0.085).

A significant increase in salivary IL-8 concentrations was observed after local probiotic supplementation (WMD: 31.82; 95% CI: 27.56, 36.08) (Fig. 5). However, a significant between-study heterogeneity was found (*I*^2^ = 72.7%, P = 0.026). Due to limited number of studies we did not perform subgroup analysis to find possible source of this heterogeneity.

In case of salivary IL-10 concentrations after local probiotic administration, no significant reduction was seen (WMD: −0.02; 95% CI: −0.10, 0.06) (Fig. 6). No evidence of between-study heterogeneity (*I*^2^ = 43.3%, P = 0.171) and publication bias (Egger’s test: 0.482) was seen.

## Disscusion

In the current meta-analysis, we found a significant increase in salivary IL-1β and IL-8 concentrations after local probiotic supplementation. However, no significant effects of oral probiotic supplementation on salivary IgA levels and also, no significant effects of local probiotic supplementation on salivary IL-6 and IL-10 concentrations were found in our meta-analysis. To the best of our knowledge, this is the first systematic review and meta-analysis summarizing the effects of oral and local probiotic supplementation on salivary immunoglobulines and cytokines.

Our findings from the current meta-analysis were in line with previous clinical trials that showed no significant increase in salivary IgA levels after oral probiotic treatments compared to placebo^5,15^. In contrast, some studies indicated a significant increase in serum IgA concentrations by probiotic consumption^1,6^. Whereas Childs *et al*. reported a significant decrease in salivary IgA concentrations after probiotic intake^2^. Although some earlier studies have shown the effect of probiotic supplementation on systemic IgA antibody releasing and B cell stimulatory activity^23,24^, the salivary concentrations of IgA, as a marker of mucosal immunity, did not influence by probiotic supplementation. This might be explained by the age of participants. Most studies have enrolled elderly people, whom antibody responses might be different from healthy middle-age adults. Moreover, saliva volume and its contents might be affected by several environmental and neural factors. Therefore, salivary levels of IgA could also be influenced by psychological and physical stress^24^. Due to limited number of publications, we were unable to do subgroup analysis by sex, age group, design and duration of trials, dose and type of probiotics. These factors may also affect our findings. It must also be taken into account that exposure to probiotics in early life through diet might also contribute to immune responses and secretion of immune-globulins in body liquids^25^.

We found a significant increase in some salivary inflammatory cytokines including IL-1β and IL-8 concentrations by local probiotic administration. However, no significant changes in IL-6 and IL-10 were observed following probiotic supplementation. These findings were in agreement with several other reports from randomized clinical trials that showed a significant increase in salivary cytokines including IL-1β^4,14^. Against to this finding, some investigators failed to find any significant effects on salivary cytokines^4,13,17^. One should keep in mind that local administration of probiotics is different from oral supplementation. The effects of local ingestion of probiotics on immune system function basically depend on individual oral biofilm environment and oral hygiene and gingival inflammation^26^. Individual oral biofilm and inflamed gums or healthy gums can differently respond to probiotic treatments. In addition, in case of gingivitis, in which we face with acute inflammation, local administration of probiotics for short-term cannot cool down inflammation due to elevated levels of inflammatory cytokines in these patients^27^. Moreover, in spite of immune-modulatory effects of local administration of probiotics and secretion to saliva, regular intake of probiotic products does not seem to be enough to initiate major alterations in oral biofilm^4^. It should also be kept in mind that the quality of primary studies can strongly influence the overall effect size. We assessed study quality in the current investigation and excluded studies with poor quality from the current analysis because of not reporting reliable effect sizes^5,6^. However, we could not perform subgroup-analysis based on quality of studies due to the limited number of publication in each area.

The possible mechanisms through which probiotic administration might affect salivary cytokines and immunoglobulines are not clearly understood. Among the possible suggested mechanisms are the effects of probiotics on increasing Treg function, through which they can induce the anti-inflammatory cytokine production, such as TGF-β, which can consequently lead to increased levels of IgA^28–31^. In addition, secretions of anti-inflammatory cytokines are up-regulated by probiotics through encouraging the anti-inflammatory M2 macrophages^32,33^.

Despite being the first meta-analysis on salivary cytokines and immunoglobulines, some limitations need to be considered. Due to limited number of publications, we were unable to do the meta-analysis on some other cytokines and immunoglobulines. The effects of probiotics are strongly dependent to age and primary exposure of host. This should be considered in the interpretation of the findings. We confined our meta-analysis to adult population and did not include studies that investigated children or adolescences. Moreover, despite the effects of salivary flow rate on the levels of salivary cytokines and immunoglobulins on one hand^34,35^ and the effect of probiotic supplementation on salivary flow rate on the other hand^12^, none of the studies had considered normalized levels of cytokines for salivary flow rate. In addition, we did not register the protocol of the current study on PROSPERO registry system due to the delay in processing the submitted protocols for studies outside the UK. This lack of registration might be a source of bias for this review. However, this review and meta-analysis was designed and performed according to the Cochrane guidelines.

In conclusion, we found that oral and local administrations of probiotics were significantly associated with increased levels of IL-1β and IL-8 in adult population. However, additional clinical trials are required to examine these effects on further pro- and anti-inflammatory cytokines and immunoglobulines.

## Supplementary information

Supplementary file. Search Strategies.
