# Supplementary material for: Effects of probiotics on salivary cytokines and immunoglobulines: a systematic review and meta-analysis on clinical trials
Source: Sci Rep. 2020 Jul 16;10:11800. doi: 10.1038/s41598-020-67037-y (PMC7366729; doi:10.1038/s41598-020-67037-y)
Supplement: Supplementary file 1 — Supplementary file. Search Strategies. [file 41598_2020_67037_MOESM1_ESM.docx]

**Effects of probiotics on salivary cytokines and immunoglobulines: a systematic review and meta-analysis on clinical trials**

**Soraiya Ebrahimpour-Koujan^1,2^, Alireza Milajerdi^2^, Bagher Larijani^3^, Ahmad Esmaillzadeh^*4,2,5^**

*^1^Students' Scientific Research Center, Tehran University of Medical Sciences, Tehran, Iran*

*^2^Department of Community Nutrition, School of Nutritional Sciences and Dietetics, Tehran University of Medical Sciences, Tehran, Iran*

*^3^Endocrinology and Metabolism Research Center, Endocrinology and Metabolism Clinical Sciences Institute, Tehran University of Medical Sciences, Tehran, Iran*

*^4^Obesity and Eating Habits Research Center, Endocrinology and Metabolism Molecular -Cellular Sciences Institute, Tehran University of Medical Sciences, Tehran, Iran*

*^5^Department of Community Nutrition, School of Nutrition and Food Science, Isfahan University of Medical Sciences, Isfahan, Iran*

**Running title:** probiotic and salivary cytokines and immunoglobulines

**Correspondence to:**

Ahmad Esmaillzadeh, PhD

Department of Community Nutrition,

School of Nutritional Sciences and Dietetics,

Tehran University of Medical Sciences,

Tehran, PO Box 14155-6117

Iran,

Tel: +98-2188955805;

Fax: +98-2188984861;

E-mail: a-[esmaillzadeh@tums.ac.ir](mailto:esmaillzadeh@tums.ac.ir)

**Funding:** No funding to report.

**Competing of interest statement:** No financial and non-financial competing of interest.

**Key words:** probiotics, lozenges, saliva, cytokine, immunoglobuline.

**Pubmed Search Strategy**

("Lactobacillus" OR "Streptococcus" OR "Saccharomyces" OR "Enterococcus" OR "Bifidobacterium" OR "Probiotic" OR "Lactococcus") AND ("Inflammation" OR "inflammatory biomarker" OR "Tumor necrosis factor" OR "C- Reactive protein" OR "Transforming growth factor beta" OR "Cytokine" OR "Acute phase reactant" OR "Matrix metalloproteinase" OR "Intercellular adhesion molecule-1" OR "Monocyte chemotactic protein 1" OR "Inflammation Mediator" OR "Adipokine" OR "Interleukin" OR "Systemic inflammation") AND ("Clinical Trial" OR "trial") AND ("saliva" OR "salivary" OR "Mouth").

**MEDLINE (ISI) Search Strategy**

(TS=("Lactobacillus") OR TS=("Streptococcus") OR TI=("Saccharomyces") OR TS=("Enterococcus") OR TS=("Bifidobacterium") OR TS=("Probiotic") OR TI=("Lactococcus")) AND (TS=("Inflammation") OR TS=("inflammatory biomarker") OR TI=("Tumor necrosis factor") OR TI=("C- Reactive protein") OR TI=("Transforming growth factor beta") OR TS=("Cytokine") OR TI=("Acute phase reactant") OR TI=("Matrix metalloproteinase") OR TI=("Intercellular adhesion molecule-1") OR TI=("Monocyte chemotactic protein 1") OR TI=("Inflammation Mediator") OR TS=("Adipokine") OR TS=("Interleukin") OR TI=("Systemic inflammation")) AND (TI=("Clinical Trial") OR TI=("trial")) AND (TS=("saliva") OR TS=("salivary") OR TI=("Mouth")).

**SCOPUS Search Strategy**

(TITLE-ABS("Lactobacillus") OR TITLE-ABS("Streptococcus") OR ALL("Saccharomyces") OR TITLE-ABS("Enterococcus") OR TITLE-ABS("Bifidobacterium") OR TITLE-ABS("Probiotic") OR ALL("Lactococcus")) AND (TITLE-ABS("Inflammation") OR ALL("inflammatory biomarker") OR ALL("Tumor necrosis factor") OR ALL("C- Reactive protein") OR ALL("Transforming growth factor beta") OR TITLE-ABS("Cytokine") OR ALL("Acute phase reactant") OR ALL("Matrix metalloproteinase") OR ALL("Intercellular adhesion molecule-1") OR ALL("Monocyte chemotactic protein 1") OR ALL("Inflammation Mediator") OR ALL("Adipokine") OR TITLE-ABS("Interleukin") OR ALL("Systemic inflammation")) AND (ALL("Clinical Trial") OR ALL("trial")) AND (TITLE-ABS("saliva") OR TITLE-ABS("salivary") OR ALL("Mouth")).

**EMBASE Search Strategy**

(ALL("Lactobacillus") OR ALL("Streptococcus") OR ALL("Saccharomyces") OR ALL("Enterococcus") OR ALL("Bifidobacterium") OR ALL("Probiotic") OR ALL("Lactococcus")) AND (ALL("Inflammation") OR ALL("inflammatory biomarker") OR ALL("Tumor necrosis factor") OR ALL("C- Reactive protein") OR ALL("Transforming growth factor beta") OR ALL("Cytokine") OR ALL("Acute phase reactant") OR ALL("Matrix metalloproteinase") OR ALL("Intercellular adhesion molecule-1") OR ALL("Monocyte chemotactic protein 1") OR ALL("Inflammation Mediator") OR ALL("Adipokine") OR TITLE-ABS("Interleukin") OR ALL("Systemic inflammation")) AND (ALL("Clinical Trial") OR ALL("trial")) AND (ALL("saliva") OR ALL("salivary") OR ALL("Mouth")).

**Google Scholar Search Strategy**

("Lactobacillus" OR "Streptococcus" OR "Saccharomyces" OR "Enterococcus" OR "Bifidobacterium" OR "Probiotic" OR "Lactococcus") AND ("Inflammation" OR "inflammatory biomarker" OR "Tumor necrosis factor" OR "C- Reactive protein" OR "Transforming growth factor beta" OR "Cytokine" OR "Acute phase reactant" OR "Matrix metalloproteinase" OR "Intercellular adhesion molecule-1" OR "Monocyte chemotactic protein 1" OR "Inflammation Mediator" OR "Adipokine" OR "Interleukin" OR "Systemic inflammation") AND ("Clinical Trial" OR "trial") AND ("saliva" OR "salivary" OR "Mouth").
